# Supplementary material for: “It’s a last straw situation”; Overdose response and preferences for seeking support from emergency services during overdose in rural Ohio
Source: PLoS One. 2026 Jan 16;21(1):e0338689. doi: 10.1371/journal.pone.0338689 (PMC12810844; doi:10.1371/journal.pone.0338689)
Supplement: Attachment — Submitted filename: p1 review_last straw_aug2025.docx [file pone.0338689.s002.docx]

Thank you for the opportunity to review this important paper. The experience and needs of rural PWUD are important and deserve further attention. This paper does that. However, there are numerous issues which need to be addressed to enhance the clarity and impact of this paper.

Overall feedback

- Overall, the paper could benefit from keen editing. Issues like consistency in formatting and tightening up of language would strengthen the communication of the paper.
- The thesis behind this study seems to imply that PWUD SHOULD call911 in the event of an OD. I am not convinced that is true and I don’t think the authors have yet made that case. They do state that SAMHSA recommends it, ok, but there are many other factors to consider, especially the wants/needs of PWUD themselves. Other authors have interrogated and discussed the complexity of these issues, and this paper would be stringer if it were to engage with that nuance and/or make a clearer argument for if and why this is the goal. (of course, in an ideal world where support was readily available and judgement free, more might be more, but that is far from the current scenario)
- The results could be strengthened by a clearer organizational structure, consistent formatting/style for presenting quotes (I prefer the indented single space style, but either way it should be implemented consistently), and editing for redundant words.
  - I also request the authors to consider editing the quotes to remove filler words that only complicate the meanings. I know folks feel differently about editing quotes, and I share a value of the importance of having participant voice come through, however, there is clearly already some editing of quote indicated by “…” so perhaps the authors can go a small step further and remove “um, like, etc.”
- The discussion needs further development. The majority of it reads like a summary and restating of the results rather than situating key findings in exiting literature and drawing interpretations and potential implications of the findings. The last few paragraphs get closer when discussing GSLs but there is plenty of room for improvement in this section

TITLE:

- I don’t actually see the title quote in the paper. Maybe I missed it? Otherwise perhaps the authors can tie that in

Abstract

- The conclusion related to rural PWID, but the paper is about PWUD in rural Ohio. I caution the authors from generalizing their findings so boldly.
  - In fact, in the paper discussion they could engage this point, on why this Ohio data may or may not be generalizable to other rural PWUD

Intro

- Many places to tighten up the writing, and the citation formatting
- Also, define PWUD at first use then use acronym consistently throughout the paper

Methods

- Overall, the methods section writing can be clarified and should be better cited. I think I can tell what process was used and I think I agree with it, but it needs to be clearer and again, cited. Especially true in the analysis subsection
  - What was the matrix used for? How was it organized? Etc.
- Was the change in consenting practices approved by IRB? If so, maybe that can be made clearer and more connected to the IRB statement.
- Are you saying you interviewed the same person twice on accident at the top of page 4 of the manuscript?! This needs more context
- I’m very confused about the decision to discuss all sorts of first responder engagement as “calling 911”. It seems calling 911 is one type of first responder outreach, and the participants describe others. Please share more about what those others are and then consider shifting the term/way of describing this to “engaging first responders” or something more umbrella.
  - This issue may be causing confusion in the way the “calling 911” term is used in the results, which comes through awkwardly and causes confusion in numerous instances

Results

- As stated above this section needs to be better organized. This may include clarifying your theme language and the use of the sub-headings. Ultimately this section needs to tell a cleaner and more accessible story of the data.
  - The second half of the results are in even more need of attention on this matter.
- In some instances, the authors use “some, many, etc.” language to indicate how salient themes were among respondents, but not always. In other places it’s a list of 1 off responses, unsynthesized. This needs work.
- Similar to the above point about some assumptions the authors may have about engaging first responders, there also emerges what seems like some assumptions that going to a hospital after an OD is “good/ideal”. This value needs to be more clearly stated and justified in the intro, or these biases in the analysis need to be otherwise addressed
- In some cases, quotes are less tied to what the author is stating as themes or bring in new themes that are unclear. The authors could review and clarify these instances.
  - One example is the quote on the bottom of p6, about guns. What do guns have to do with the saving folks from OD?
  - On the bottom of the next page, redacted needs to be clarified, not with names of course, but is it referencing the police or the partner or... who?
- Define less commonly understood concepts for the reader
  - Ex. Misdemeanor crime in intro; Trap house in results
- Is fear of legal consequences not very related to legal status? Could these sections be connected or somehow in discussion with each other?

Discussion

- The authors use the term “pragmatic” quite a bit to make sense of the findings. I suggest defining this term as being used here
- See above for larger picture concerns with the discussion
