# Supplementary material for: “It’s a last straw situation”; Overdose response and preferences for seeking support from emergency services during overdose in rural Ohio
Source: PLoS One. 2026 Jan 16;21(1):e0338689. doi: 10.1371/journal.pone.0338689 (PMC12810844; doi:10.1371/journal.pone.0338689)
Supplement: Attachment — Submitted filename: Responses to Reviewers_LastStraw PLos.docx [file pone.0338689.s004.docx]

Response to Reviewers: “It’s a last straw situation”; Overdose Response and Preferences for Seeking Support from Emergency Services during Overdose in Rural Ohio.

Dear editorial team at PLOS ONE and Reviewers,

Thank you to the editorial team and reviewers for the thoughtful feedback and opportunity to revise our manuscript related to overdose response and decisions to seek support from first responders in Appalachian Ohio. We have amended the manuscript accordingly. Please find responses to your questions or recommendations in red text, below. Specific additions to the text have also been included when appropriate, though some of the larger organizational changes that were recommended were only included in the body of the manuscript due to length. We thank the reviewers for their time and effort. The reviews have improved the manuscript.

Sincerely,

David Colston

Reviewer #1: The authors present a paper titled “It’s a last straw situation”; Overdose Response and Preferences for Seeking Support from Emergency Services during Overdose in Rural Ohio.
This is an important qualitative study that explores a crucial, underexamined area: the real-world dynamics and decision-making processes behind whether people who use drugs (PWUD) in rural Appalachia call 911 during overdose events. The manuscript is well-organized, thoughtfully written, and grounded in strong public health relevance. With some strengthening of methods transparency, clearer demographic framing, and more focused theme organization, this paper will be an important contribution.

Here are some comments:

1. Introduction: Emphasize the unique contribution of this paper to make it clearer upfront in the Introduction. What gap in knowledge does this study fill that others don’t? Why is this particular rural Ohio context novel?

We have added information regarding what contribution this paper will make to the broader literature in the introduction, specifically as it relates to understanding engagement of first responders during overdose in Appalachia, and why Ohio is the appropriate setting for this study.

To reduce drug-related morbidity and mortality in Appalachia, further research is needed to understand how PWUD interact with first responders during an overdose and what motivates them to do so. Ohio, home to 32 Appalachian counties [1], offers an ideal study setting to explore these decision making-processes given its historical experience with pill mills (clinics that overly prescribed opioids in early stages of the overdose epidemic)[2]. Moreover, although overdose mortality rates in Ohio declined significantly from 2024 to 2025 mirroring national trends [3], they remain alarmingly high [3, 4]. (Page 3, Lines 84-91).

2. Method: Regarding coding - how many coders were involved? Was intercoder reliability assessed? How were disagreements resolved?

We have added a sentence to the methods section explaining that all coding was conducted by one person. Because there were not multiple coders, intercoder reliability was not assessed. We have also added sentences on the role of CB and VFG in reviewing codes, themes, and how excerpts fit together. Modifications were then made at later coding stages and in manuscript preparation.

“All coding was conducted by one researcher (DCC).” (Page 6, Lines 137-138)

“Themes and related excerpts were shared with members of the research team (CB, VFG) at various stages of analysis. Feedback from CB and VFG was incorporated into DCC’s analytic approach, framing, and interpretation of the data.” (Page 6, Lines 140-143)

Regarding results - the manuscript tells us N = 44, but gives very little demographic context (e.g., age, gender, race, legal status, housing status). This weakens interpretation of findings, especially since issues like stigma, criminal-legal involvement, and healthcare access are highly intersectional. Please provide a summary table of participant characteristics to help contextualize the quotes and themes.

We did not systematically collect demographic data on race/ethnicity or criminal-legal involvement, which we acknowledge as a significant limitation Accordingly we added a limitations section to explicitly address this issue and call for further research on the intersection of race and ethnicity, legal system involvement, and the use of harm reduction strategies that may increase exposure to judgment, violence, or arrest.

“This study has several limitations. First, we did not collect demographic data on race and ethnicity or involvement in the legal system among interviewed participants. We therefore cannot address the role of race/ethnicity on the role of fear for arrest, the risk of being detained due to warrants in neighboring counties, and experienced racism in the judicial system. We strongly urge more intersectional research [5] to better understand how holding multiple marginalized identities – including being a person who uses drugs – shapes decisions to seek help from first responders during an overdose or or to engage in stigmatized overdose prevention efforts.”(Page 17, Lines 416-422)

3. Discussion: Some recommendations—e.g., “ensure law enforcement doesn’t arrest people at the scene”—while valid (and policy-oriented) could be hard to implement without more nuance. I suggest expanding on what practical steps could improve GSL awareness, reduce stigma among EMS/police, and build trust. Could peer responders or harm reduction outreach workers play a role?

In addition to our existing text situating the literature on ways to build trust in the seeking support during an overdose and how naloxone trainings can be received and improve knowledge for proper handling of an overdose response among law enforcement, we have added possible ways to increase awareness of the GSL.

“Fear of arrest appears to be minimally alleviated by Ohio’s Good Samaritan Law (GSL), which offers protections for those seeking help during an overdose. PWUD are commonly unaware of the GSL or its provisions, though findings differ across studies [6-10]. According to the Social Cognitive Theory, awareness and accurate understanding of the protections offered by GSL are prerequisites for making informed decisions [11, 12]. The best approach to raise awareness of the GSL among PWUD is unclear, but the demonstrated connectivity and information-sharing around risk-reduction practices within PWUD networks [13] may offer a promising approach. “Champions” may also be a promising approach, as it has been effective in implementing health services [14]. PWUD have played an important role in distributing peer support services for people in recovery [15, 16] and sharing resources, such as naloxone, to reduce the risk of injury or death from drug use [13, 17, 18]. Consequently, they are uniquely positioned to increase awareness for GSLs, especially provided with adequate training to ensure accurate knowledge [16, 19].” (Page 14, Lines 353-365).

4. Other minor comments: The title is compelling, but the phrase “last straw situation” is buried in the text and not highlighted much. Either elevate its significance in the narrative or consider a more thematically appropriate title.

Thank you for this comment. We have added a sub-heading in the results and pulled the “last straw” quote into the top of the discussion section where we briefly summarize primary study findings, and the conclusion.

In the Results:
***Seeking Support from First Responders as a “Last Straw” during Overdose
“***Most participants preferred to seek support from first responders only as a “a last straw situation,” that is, if lay-responders could not revive the victims on their own.” (Page 7, Lines 174-175)

In the Discussion:
“Pragmatism was rooted in the confidence of their overdose reversal abilities, with many only seeking help as a “last straw” – unable to revive the victim on their own.” (Page 13, Lines 339-340)

In the Conclusion:

In rural Appalachian Ohio, we observed that seeking support from first responders during an overdose was a “last straw situation”, although some barriers to seeking support are reversible. (Page 17, Lines 428-430)

Also, the data availability note says data access is restricted due to human subject protections. This is reasonable, but if possible, consider sharing a codebook or de-identified quote bank for transparency.

We have included a subset of the matrix used to analyze themes across and within participants’ experiences and stated preferences regarding seeking support from first responders during overdose. The excerpts presented are from a subset of study participants and are intended to provide a clearer understanding of the data and structure of our analysis. Those interested in further analyzing the data can contact me directly at [dcolston@ad.unc.edu](mailto:dcolston@ad.unc.edu) or Principal Investigators Vivian F. Go or William C. Miller. We will follow appropriate channels to facilitate access.

Thank you for the opportunity to review this important paper. The experience and needs of rural PWUD are important and deserve further attention. This paper does that. However, there are numerous issues which need to be addressed to enhance the clarity and impact of this paper.

Overall feedback

- Overall, the paper could benefit from keen editing. Issues like consistency in formatting and tightening up of language would strengthen the communication of the paper.

We made considerable edits throughout the paper to improve clarity. We removed redundant and extraneous excerpts that detracted from the paper’s focus. We also streamlined much of the discussion section to ensure that findings are either compared to the broader literature base or connected to implications for policy and future intervention efforts.

- The thesis behind this study seems to imply that PWUD SHOULD call911 in the event of an OD. I am not convinced that is true and I don’t think the authors have yet made that case. They do state that SAMHSA recommends it, ok, but there are many other factors to consider, especially the wants/needs of PWUD themselves. Other authors have interrogated and discussed the complexity of these issues, and this paper would be stringer if it were to engage with that nuance and/or make a clearer argument for if and why this is the goal. (of course, in an ideal world where support was readily available and judgement free, more might be more, but that is far from the current scenario)

Thank you for this comment, we agree, and have added the following sections in the discussion and conclusion:.

Discussion:

“The demonstrated success PWUD have had in reviving people who have overdosed, the perceived risks experts ask them to take on, and the context-specific decision-making required in each case suggest we should critically reconsider the blanket recommendation to seek support from first responders during an overdose. While ideal in theory, it is not always practical in reality. This underscores the importance of free and widely accessible access to naloxone for PWUD and community members enabling them to effectively intervene when an overdose is witnessed” (Page 17, Lines 415-421)

Conclusion:

“Recognition must also be given to the countless lives saved by PWUD, who often reverse overdoses without assistance from first responders. Given their success in doing so, and the fear of stigma or arrest that may result from a 9-1-1 call, we strongly advocate for the continued accessibility of affordable naloxone to laypersons (non-first responders) and PWUD.” (Page 18, Lines 439-443)

- The results could be strengthened by a clearer organizational structure, consistent formatting/style for presenting quotes (I prefer the indented single space style, but either way it should be implemented consistently), and editing for redundant words.
  - I also request the authors to consider editing the quotes to remove filler words that only complicate the meanings. I know folks feel differently about editing quotes, and I share a value of the importance of having participant voice come through, however, there is clearly already some editing of quote indicated by “…” so perhaps the authors can go a small step further and remove “um, like, etc.”

We have edited the results section for brevity, removed extraneous or unnecessarily repetitive quotes, and edited filler words out of quotes wherever possible without altering the meaning.

Regarding formatting, we have indented quotes longer than 3 lines, as well as instances where multiple short quotes are used to succinctly encapsulate the highlighted theme.

- The discussion needs further development. The majority of it reads like a summary and restating of the results rather than situating key findings in exiting literature and drawing interpretations and potential implications of the findings. The last few paragraphs get closer when discussing GSLs but there is plenty of room for improvement in this section

We reworked the discussion to integrate our major study findings within the existing literature, interpret findings further, and highlight opportunities for policy and possible intervention efforts.

TITLE:

- I don’t actually see the title quote in the paper. Maybe I missed it? Otherwise perhaps the authors can tie that in

We added a sub-heading in the results and pulled the “last straw” quote to the top of the discussion section where we briefly summarize primary study findings, and the conclusion.

In the Results:
***Seeking Support from First Responders as a “Last Straw” during Overdose
“***Most participants preferred to seek support from first responders only as a “a last straw situation,” that is, if lay-responders could not revive the victims on their own.” (Page 7, Lines 174-175)

In the Discussion:
“Pragmatism was rooted in the confidence of their overdose reversal abilities, with many only seeking help as a “last straw” – unable to revive the victim on their own.” (Page 13, Lines 339-340)

In the Conclusion:

In rural Appalachian Ohio, we observed that seeking support from first responders during an overdose was a “last straw situation”, although some barriers to seeking support are reversible. (Page 17, Lines 428-430)

Abstract

- The conclusion related to rural PWID, but the paper is about PWUD in rural Ohio. I caution the authors from generalizing their findings so boldly.
  - In fact, in the paper discussion they could engage this point, on why this Ohio data may or may not be generalizable to other rural PWUD

We have amended the conclusion in the abstract to make it clear results are from rural Ohio, and likely are not generalizable to the entire United States, but may be generalizable to other rural, Appalachian areas.

**Significance/Contribution**: This study details intervenable factors that could prevent overdoses from escalating to fatalities in rural Ohio, including: increasing awareness and knowledge for Good Samaritan Laws to encourage calls to 9-1-1 during overdose, ensuring unlawful arrests are not made at the scene of an overdose, and de-stigmatizing substance use among first responders and health care professionals. Findings may not be generalizable to the entire United States, but may have applicability in other rural, Appalachian areas. (Page 1, Lines 37-42)

We have also taken your suggestion and added a section in the discussion section related to the importance in the context of Appalachia and generalizability.

*“Regional Relevance and Generalizability*

“*Regional Relevance and Generalizability*

The pragmatic decision made by many to only seek support for overdose victims as a “last straw” speaks to the broader belief that these PWUD from rural Ohio can take care of themselves and each other – a tenet central to the cultural ideology of Appalachia that values both self-reliance and collectivism among kin [20]. In this way, findings from our study – while likely not fully generalizable to the entire United States – may be particularly relevant to PWUD in other Appalachian towns. Moreover, takeaways for improving behavioral health may have broader applicability beyond this region, as many PWUD go out of their way to care for one another, often serving as the first line of defense in overdose response and revival [17].” (Pages 16, 401-408)

Intro

- Many places to tighten up the writing, and the citation formatting

We tightened up the writing, deleting redundant and distracting sentences in the Introduction. We also re-formatted the citations according to PLOS ONE guidelines.

- Also, define PWUD at first use then use acronym consistently throughout the paper

We have amended the manuscript to ensure PWUD is used throughout after it is first established in the introduction.

Methods

- Overall, the methods section writing can be clarified and should be better cited. I think I can tell what process was used and I think I agree with it, but it needs to be clearer and again, cited. Especially true in the analysis subsection

We have added the following to clarify methods used, including the number of coders, the involvement of other research team members, an additional citation related to our iterative analytic approach [21] and initial code development [22], and organization of matrices.

Related to coders and team involvement:

“All coding was conducted by one researcher (DCC).” (Page 6, Lines 137-138)

“Themes and related excerpts were shared with members of the research team (CB, VFG) at various stages of analysis. Feedback from CB and VFG was incorporated into DCC’s analytic approach, framing, and interpretation of the data.” (Page 6, Lines 140-143)

- What was the matrix used for? How was it organized? Etc.

We added the following text to improve clarification of our matrix:

“Matrices included quotes by participant to allow for comparison of personal overdose experiences, witnessed overdoses, seeking support from first responders or healthcare providers during personal or witnessed overdose, preferences for calling 9-1-1 during an overdose, rationale for preference, and general attitudes or experiences with first responders or law enforcement.” (Page 6, Lines 143-147) and reasoning:

“Organizing matrices by theme and participant allowed us to analyze passages by comparing like and contrasting themes between participants and in the context of the participant’s entire interview. We took this strategy because, with the exception of those firmly closed to seeking support from first responders during an overdose, participants rarely stated a clear preference for or against, and those that did express a preference often qualified their statement or provided accounts or perceptions that ran contrary to their initially stated preference. As such, coding participant preferences for or against seeking first responder support during an overdose was more interpretive, and based on their interview as a whole rather than one isolated quote. We also were interested in factors that may have preceded and contributed to their preferences for/against seeking support from first responders.”

(Page 6-7, Lines 154-163)

We have included a subset of the matrix used to analyze themes across and within participants’ experiences and stated preferences regarding seeking support from first responders during overdose. The excerpts presented are from a subset of study participants and are intended to provide a clearer understanding of the data and structure of our analysis. Those interested in further analyzing the data can contact me directly at [dcolston@ad.unc.edu](mailto:dcolston@ad.unc.edu) or Principal Investigators Vivian F. Go or William C. Miller. We will follow appropriate channels to facilitate access.

- Was the change in consenting practices approved by IRB? If so, maybe that can be made clearer and more connected to the IRB statement.

We included a sentence explaining the IRB approved changes to our study related to COVID-19.

“The Ohio State IRB was notified and approved of changes in the method of data collection.” (Page 5, Lines 116-117).

- Are you saying you interviewed the same person twice on accident at the top of page 4 of the manuscript?! This needs more context

Thank you for the opportunity to provide more context. Given multiple interviewers conducting interviews at different times, one individual was inadvertently interviewed twice. We have added the following text to provide additional context to readers:

“We discarded interviews due to poor audio quality (n=2) and a duplicate interview that occurred due to having multiple interviewers conducting interviews at different times (n=1). We discovered the repeat-participant when reviewing transcripts, as the participant recounted the same story and individuals in the story by name. “ (Page 5, Lines 120-123)

- I’m very confused about the decision to discuss all sorts of first responder engagement as “calling 911”. It seems calling 911 is one type of first responder outreach, and the participants describe others. Please share more about what those others are and then consider shifting the term/way of describing this to “engaging first responders” or something more umbrella.
  - This issue may be causing confusion in the way the “calling 911” term is used in the results, which comes through awkwardly and causes confusion in numerous instances

In response to your recommendation, we have removed this section from the methods and no longer use ‘calling 911’ as blanket terminology throughout the manuscript. Instead, when generalizing, we use “seek support from first responders” to be more accurate, though there are instances where keeping the terminology of ‘calling 9-1-1’ made sense (in context and in some instances to avoid repetitiveness).

Results

- As stated above this section needs to be better organized. This may include clarifying your theme language and the use of the sub-headings. Ultimately this section needs to tell a cleaner and more accessible story of the data.
  - The second half of the results are in even more need of attention on this matter.
- In some instances, the authors use “some, many, etc.” language to indicate how salient themes were among respondents, but not always. In other places it’s a list of 1 off responses, unsynthesized. This needs work.

We have further synthesized study findings, cutting extraneous quotes that fit the broader theme and summarizing more where possible. Descriptors like ‘many’ are still used to showcase the frequency across the sample, while one-off quotes are only described as ‘one person’ in the text if their statement added a particularly novel or slightly different contribution related to a highlighted theme.

- Similar to the above point about some assumptions the authors may have about engaging first responders, there also emerges what seems like some assumptions that going to a hospital after an OD is “good/ideal”. This value needs to be more clearly stated and justified in the intro, or these biases in the analysis need to be otherwise addressed

This was not our intention, rather we aimed to juxtapose what many organizations, such as SAMHSA, recommend as the ‘gold standard’ with the reality on the ground. We have tempered language that may come across as paternalistic, and added a more extensive discussion about re-thinking blanket statements (such as the recommendation that 9-1-1 should always be called) which are often impractical in real-world contexts (see responses above and below for the added manuscript text). We would be happy to include a positionality statement if needed.

- In some cases, quotes are less tied to what the author is stating as themes or bring in new themes that are unclear. The authors could review and clarify these instances.
  - One example is the quote on the bottom of p6, about guns. What do guns have to do with the saving folks from OD?
  - On the bottom of the next page, redacted needs to be clarified, not with names of course, but is it referencing the police or the partner or... who?

We agree that the discussion related to guns (as well as some other quotes that we initially felt useful for context) detracted from the overall purpose of the study. We have deleted the quotes about guns, violence at the hands of police, judiciary issues related to their partner’s domestic violence, etc.

- Define less commonly understood concepts for the reader
  - Ex. Misdemeanor crime in intro; Trap house in results

We have added descriptions for “trap house” and “shooting gallery” and describe potentially less understood topics:

“Location was one barrier to seeking support during an overdose: whether at a trap house (locations drugs are sold) or shooting gallery (shared space where drugs are used) [23, 24]”(Page 11, Lines 277-278)

- Is fear of legal consequences not very related to legal status? Could these sections be connected or somehow in discussion with each other?

We did not collect information on legal status. We have addressed this by adding a Limitations section (below) calling for more research:

*“Limitations*

This study has several limitations. First, we did not collect demographic data on race and ethnicity or involvement in the legal system among interviewed participants. We therefore cannot address the role of race/ethnicity on the role of fear for arrest, the risk of being detained due to warrants in neighboring counties, and experienced racism in the judicial system. We strongly urge more intersectional research [5] to better understand how holding multiple marginalized identities – including being a person who uses drugs – shapes decisions to seek help from first responders during an overdose or or to engage in stigmatized overdose prevention efforts.”

(Page 17, Lines 415-422)

Discussion

- The authors use the term “pragmatic” quite a bit to make sense of the findings. I suggest defining this term as being used here
- See above for larger picture concerns with the discussion

We have used your recommendations to substantially revise the discussion, situating findings within the broader literature, deepening the interpretation of analytic results, and incorporating recommendations for both interventions and policy.

We have added a brief definition of pragmatic: “We found in rural, Appalachian Ohio, PWUD were largely pragmatic, or “practical as opposed to idealistic” [25], in their decisions to seek support from first responders during overdose, consistent with some [26, 27] but not all previously published studies [28].” (Pages 13, Lines 336-340) and carried the concept of ‘idealistic vs. practical’ throughout the discussion to emphasize the successes reversing overdoses without first responder help as there is not a one-size fits all approach.

**References**

1.	Foundation for Appalachian Ohio. 32 County Region of Appalachain Ohio: Foudnation for Appalachian Ohio; [cited 2025 10/23]. Available from: <https://appalachianohio.org/wp-content/uploads/2022/06/32-County-Map.pdf>.

2.	Schalkoff CA, Lancaster KE, Gaynes BN, Wang V, Pence BW, Miller WC, et al. The Opioid and Related Drug Epidemics in Rural Appalachia: A Systematic Review of Populations Affected, Risk Factors, and Infectious Diseases. Substance Abuse. 2020;41(1):35-69. doi: 10.1080/08897077.2019.1635555. PubMed PMID: 31403903.

3.	Provisional drug overdose death counts. [Internet]. National Center for Health Statistics 2025 [cited September 10, 2025]. Available from: <https://www.cdc.gov/nchs/nvss/vsrr/drug-overdose-data.htm>

4.	National Center for Health Statistics. Drug Overdose Mortality. National Center for Health Statistics, 2025.

5.	Hall OT, Trimble C, Garcia S, Grayson S, Joseph L, Entrup P, et al. Who feels safe calling 911: are prior experiences of anti-Black racial discrimination associated with hesitancy seeking emergency medical services in the event of accidental drug overdose? – a study protocol. Annals of Medicine. 2025;57(1):2439540. doi: 10.1080/07853890.2024.2439540.

6.	Schneider KE, Park JN, Allen ST, Weir BW, Sherman SG. Knowledge of Good Samaritan Laws and Beliefs About Arrests Among Persons Who Inject Drugs a Year After Policy Change in Baltimore, Maryland. Public Health Rep. 2020;135(3):393-400. Epub 20200407. doi: 10.1177/0033354920915439. PubMed PMID: 32264789; PubMed Central PMCID: PMCPMC7238711.

7.	Schneider KE, Park JN, Allen ST, Weir BW, Sherman SG. Knowledge of Good Samaritan Laws and Beliefs About Arrests Among Persons Who Inject Drugs a Year After Policy Change in Baltimore, Maryland. Public Health Reports. 2020;135(3):393-400. doi: 10.1177/0033354920915439. PubMed PMID: 32264789.

8.	Banta-Green CJ, Beletsky L, Schoeppe JA, Coffin PO, Kuszler PC. Police Officers’ and Paramedics’ Experiences with Overdose and Their Knowledge and Opinions of Washington State’s Drug Overdose–Naloxone–Good Samaritan Law. Journal of Urban Health. 2013;90(6):1102-11. doi: 10.1007/s11524-013-9814-y.

9.	Carroll JJ, Mital S, Wolff J, Noonan RK, Martinez P, Podolsky MC, et al. Knowledge, preparedness, and compassion fatigue among law enforcement officers who respond to opioid overdose. Drug and Alcohol Dependence. 2020;217:108257. doi: <https://doi.org/10.1016/j.drugalcdep.2020.108257>.

10.	Footer KHA, Urquhart GJ, Silberzahn B, Rouhani S, Weicker NP, Owczarzak J, et al. PWUD Experiences of Criminal Justice Reform: Enduring Tensions Between Policing and Harm Reduction in Baltimore, MD. Contemporary Drug Problems. 2023;50(2):176-89. doi: 10.1177/00914509221136913.

11.	Bandura A. Health promotion by social cognitive means. Health Educ Behav. 2004;31(2):143-64. doi: 10.1177/1090198104263660. PubMed PMID: 15090118.

12.	Bandura A. Social foundations of thought and action: A social cognitive theory. Englewood Cliffs, NJ, US: Prentice-Hall, Inc; 1986. xiii, 617-xiii, p.

13.	Ibragimov U, Cooper KE, Batty E, Ballard AM, Fadanelli M, Gross SB, et al. Factors that influence enrollment in syringe services programs in rural areas: a qualitative study among program clients in Appalachian Kentucky. Harm Reduction Journal. 2021;18(1):68. doi: 10.1186/s12954-021-00518-z.

14.	Santos WJ, Graham ID, Lalonde M, Demery Varin M, Squires JE. The effectiveness of champions in implementing innovations in health care: a systematic review. Implementation Science Communications. 2022;3(1):80. doi: 10.1186/s43058-022-00315-0.

15.	Jacobson N, Trojanowski L, Dewa CS. What do peer support workers do? A job description. BMC Health Services Research. 2012;12(1):205. doi: 10.1186/1472-6963-12-205.

16.	Peng L, Stack E, Cooke A, Hartzler B, Cook R, Leichtling G, et al. Centering peers in design and training for a peer-delivered contingency management program for self-identified harm reduction and treatment goals. Harm Reduct J. 2025;22(Suppl 1):72. Epub 20250506. doi: 10.1186/s12954-025-01213-z. PubMed PMID: 40329274; PubMed Central PMCID: PMCPMC12057027.

17.	Rochester E, Graboyes M. Experiences of people who use drugs with naloxone administration: a qualitative study. Drugs: Education, Prevention and Policy. 2022;29(1):54-61. doi: 10.1080/09687637.2020.1855116.

18.	Ashford RD, Curtis B, Brown AM. Peer-delivered harm reduction and recovery support services: initial evaluation from a hybrid recovery community drop-in center and syringe exchange program. Harm Reduct J. 2018;15(1):52. Epub 20181022. doi: 10.1186/s12954-018-0258-2. PubMed PMID: 30348170; PubMed Central PMCID: PMCPMC6198436.

19.	Sisson LN, Winiker AK, Triece T, Rousch RS, Rouhani S, Owczarzak J, et al. "All You Can Do is What You Know to Do": Naloxone Knowledge and Uncertainty Among People Who Use Drugs in Maryland amid a Volatile Drug Market. Subst Use Misuse. 2025;60(8):1164-72. Epub 20250412. doi: 10.1080/10826084.2025.2491768. PubMed PMID: 40219904; PubMed Central PMCID: PMCPMC12199276.

20.	Treadway J. Who are your people? Investigating collectivism in Appalachia. Athens, GA: University of Georgia; 2014.

21.	Srivastava P, Hopwood N. A Practical Iterative Framework for Qualitative Data Analysis. International Journal of Qualitative Methods. 2009;8(1):76-84. doi: 10.1177/160940690900800107.

22.	Deterding NM, Waters MC. Flexible Coding of In-depth Interviews: A Twenty-first-century Approach. Sociological Methods & Research. 2021;50(2):708-39. doi: 10.1177/0049124118799377.

23.	Celentano DD, Vlahov D, Cohn S, Anthony JC, Solomon L, Nelson KE. Risk factors for shooting gallery use and cessation among intravenous drug users. American Journal of Public Health. 1991;81(10):1291-5. doi: 10.2105/ajph.81.10.1291. PubMed PMID: 1928528.

24.	Tobin KE, Davey-Rothwell M, Latkin CA. Social-Level Correlates of Shooting Gallery Attendance: A Focus on Networks and Norms. AIDS and Behavior. 2010;14(5):1142-8. doi: 10.1007/s10461-010-9670-7.

25.	Merriam-Webster. pragmatic.

26.	Koester S, Mueller SR, Raville L, Langegger S, Binswanger IA. Why are some people who have received overdose education and naloxone reticent to call Emergency Medical Services in the event of overdose? Int J Drug Policy. 2017;48:115-24. Epub 20170719. doi: 10.1016/j.drugpo.2017.06.008. PubMed PMID: 28734745; PubMed Central PMCID: PMCPMC5825210.

27.	Lankenau SE, Wagner KD, Silva K, Kecojevic A, Iverson E, McNeely M, et al. Injection Drug Users Trained by Overdose Prevention Programs: Responses to Witnessed Overdoses. Journal of Community Health. 2013;38(1):133-41. doi: 10.1007/s10900-012-9591-7.

28.	Moallef S, Choi J, Milloy MJ, DeBeck K, Kerr T, Hayashi K. A drug-related Good Samaritan Law and calling emergency medical services for drug overdoses in a Canadian setting. Harm Reduct J. 2021;18(1):91. Epub 20210826. doi: 10.1186/s12954-021-00537-w. PubMed PMID: 34446026; PubMed Central PMCID: PMCPMC8393452.
